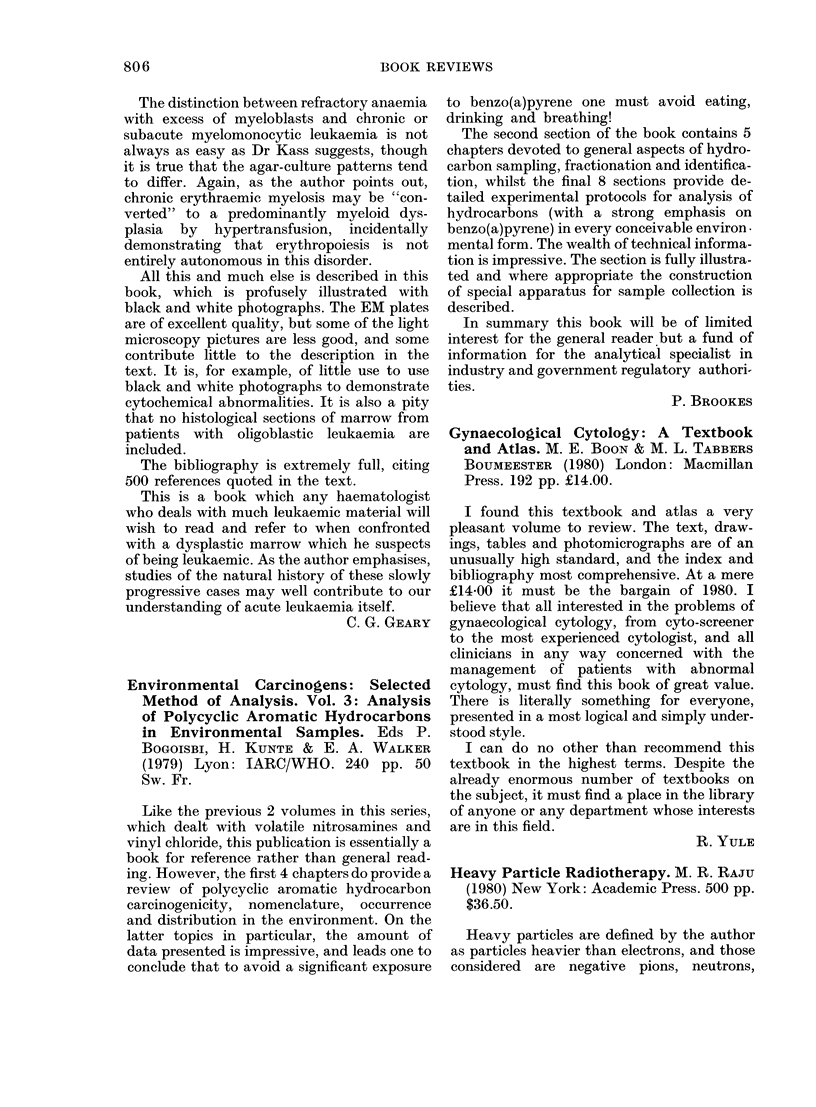# Environmental Carcinogens: Selected Method of Analysis. Vol. 3: Analysis of Polycyclic Aromatic Hydrocarbons in Environmental Samples

**Published:** 1980-11

**Authors:** P. Brookes


**Environmental Carcinogens: Selected Method of Analysis. Vol. 3: Analysis of Polycyclic Aromatic Hydrocarbons in Environmental Samples.** Eds P. BOGOISBI, H. KUNTE & E. A. WALKER (1979) Lyon: IARC/WHO. 240 pp. 50 Sw. Fr.

Like the previous 2 volumes in this series, which dealt with volatile nitrosamines and vinyl chloride, this publication is essentially a book for reference rather than general reading. However, the first 4 chapters do provide a review of polycyclic aromatic hydrocarbon carcinogenicity, nomenclature, occurrence and distribution in the environment. On the latter topics in particular, the amount of data presented is impressive, and leads one to conclude that to avoid a significant exposure to benzo(a)pyrene one must avoid eating, drinking and breathing!

The second section of the book contains 5 chapters devoted to general aspects of hydrocarbon sampling, fractionation and identification, whilst the final 8 sections provide detailed experimental protocols for analysis of hydrocarbons (with a strong emphasis on benzo(a)pyrene) in every conceivable environmental form. The wealth of technical information is impressive. The section is fully illustrated and where appropriate the construction of special apparatus for sample collection is described.

In summary this book will be of limited interest for the general reader but a fund of information for the analytical specialist in industry and government regulatory authorities.

P. BROOKES